# *Trypanosoma cruzi* SSP4 Amastigote Protein Induces Expression of Immunoregulatory and Immunosuppressive Molecules in Peripheral Blood Mononuclear Cells

**DOI:** 10.1155/2012/829139

**Published:** 2012-11-01

**Authors:** Yadira Morán-Utrera, Aracely López-Monteon, José Luis Rosales-Encina, Enrique Méndez-Bolaina, Angel Ramos-Ligonio

**Affiliations:** ^1^LADISER Inmunología y Biología Molecular, Facultad de Ciencias Químicas, Orizaba, VER, Mexico; ^2^Centro de Investigaciones Biomédicas, Universidad Veracruzana, Xalapa, VER, Mexico; ^3^Laboratorio de Biología Molecular, Departamento de Infectómica y Patogénesis Molecular, Centro de Investigaciones y de Estudios Avanzados del Instituto Politécnico Nacional, Mexico City, DF, Mexico; ^4^Facultad de Ciencias Químicas, Universidad Veracruzana, 94340 Orizaba, Veracruz, Mexico

## Abstract

The acute phase of Chagas' disease in mice and human is marked by states of immunosuppression, in which *Trypanosoma cruzi* replicates extensively and releases immunomodulatory molecules that delay parasite-specific responses mediated by effector T cells. This mechanism of evasion allows the parasite to spread in the host. Parasite molecules that regulate the host immune response during Chagas' disease have not been fully identified, particularly proteins of the amastigote stage. In this work, we evaluated the role of the GPI anchored SSP4 protein of *T. cruzi* as an immunomodulatory molecule in peripheral blood mononuclear cells (PBMCs). rMBP::SSP4 protein was able to stimulate nitric oxide (NO) production. Likewise, rMBP::SSP4 induced the expression of genes and production of molecules involved in the inflammatory process, such as, cytokines, chemokines, and adhesion molecules (CAMs) as determined by RT-PCR and ELISA. These results suggest that the amastigote SSP4 molecule could play a key role in the immunoregulatory and/or immunosuppressive process observed in the acute phase of infection with *T. cruzi*.

## 1. Introduction

Chagas' disease is a zoonosis caused by the protozoan parasite *Trypanosoma cruzi*, and it is a major public health problem in most of Latin America and in particular in Mexico. Indeed, the WHO estimates that about 8–11 million persons are infected worldwide [1]. *T. cruzi* infects many cell types, including myocytes, fibroblast, vascular endothelial, and smooth muscle cells among other cells. Since the monocyte is a target cell in *T. cruzi* infection and monocytes play a major role in regulating immune responses, monocyte dysfunction may contribute to host immunosuppression [2]. It has been observed that during the experimental infection with *T. cruzi*, there is an increased expression of proinflammatory mediators, including cytokines [3], chemokines [4], vascular adhesion molecules [5], and nitric oxide synthase [6] among other molecules [7], which promotes the inflammatory process and vascular damage. There is evidence that immune mechanisms are involved in the pathogenesis of many parasitic infections. The initial stages of the disease are generally characterized by the induction of a nonspecific lymphoproliferation, which is believed to disrupt antigen recognition and interfere with protective immune responses. Paradoxically, in most cases, a state of immunosuppression can be evidenced. This hyporesponsiveness to antigen-specific and polyclonal stimuli in chronic parasitic infections could be related to immunosuppressive cytokines secreted by antigen presenting cells and regulatory T cells. A growing list of parasite-derived molecules able to exert immunomodulatory activities on the cells of the immune system leading to such polarized cytokine secretion has been reported [8].

The intracellular phase of the parasite has been poorly studied, and it is known that *T. cruzi* amastigote surface antigens induce an immune response [9, 10]. However, few such molecules have been thoroughly studied. Recently our group has studied a *T. cruzi* amastigote-specific surface protein (SSP4), that is bound to the plasma membrane by a GPI anchor, which is released to the culture medium by phospholipase C activity [11]. The gene for this protein (cDNA) was cloned and partially characterized [12], obtaining the recombinant protein rMBP::SSP4. We have reported that this protein is a modulator of humoral and cellular immune responses in murine model, inducing low levels of IgA, IgM, and IgG3, but high levels of IgG1, IgG2a, and IgG2b isotypes; moreover, it is able to modulate nitric oxide production, as well as, to modulate the expression of cytokines *in vivo* in murine macrophages after immunization [13], suggesting that rMBP::SSP4 might exert a regulatory influence on macrophages during the immune response against *T. cruzi.* Also, it has been observed that the protein, rMBP::SSP4 activates a population of IL-10/IFN-*γ*-secreting CD4+ T cells [10], which has been observed to be activated during chronic infections and is responsible for prolonged persistence of parasite and for host protection against severe inflammatory responses [14]. Finally, it was observed that immunization with rMBP::SSP4 protein makes mice more susceptible to trypomastigote infection, with high mortality rates, whereas mice immunized with a eukaryotic expression plasmid containing the rMBP::SSP4 cDNA were able to control the acute phase of infection [15]. This suggests that the SSP4 antigen plays a role in the infection process. It should be noted that parasite molecules that regulate the host (human) immune response during Chagas' disease have not been fully identified, and to date there are few reports about the role of amastigote proteins in the development of the disease; therefore, it is important to characterize parasite molecules and their involvement in the pathology of the disease.

In this work, we analyzed the effect of rMBP::SSP4, a recombinant protein derived from T. cruzi in cursive (a major surface glycoprotein (SSP4) that is bound to the plasma membrane by a GPI anchor) on the induction of nitric oxide (NO), cytokines, chemokines, and adhesion molecules (CAMs) using humans' PBMC.

## 2. Materials and Methods

### 2.1. MBP and rMBP::SSP4 Expression and Purification

The conditions of purification of the recombinant protein rMBP::SSP4 were previously reported [13]. Briefly, the TcSSP4 (GeneBank, EMBL, and DDJ databases accession number AF480943) was cloned in the *Eco* RI site of the expression vectors pMAL-C2 (New England BioLabs) resulting in the plasmid pMALSSP4. This plasmid and plasmid pMALC2 were used to transform *E. coli* DH5-*α*, to obtain the fusion proteins rMBP::SSP4 and maltose binding protein (MBP), which were induced and purified according to the manufacturer. Either MBP or MBP-fusion protein were eluted by competition with free maltose (10 mM maltose in 20 mM Tris-HCl pH 7.4, 200 mM NaCl, and 1 mM EDTA), and then acetone-precipitated. Protein purification was analyzed by 10% SDS-PAGE in reducing conditions and coomassie blue staining [16].

### 2.2. PBMC Isolation

Heparinized fresh human whole blood (10 IU heparin/mL) was diluted 1 : 2 with PBS (137 mM NaCl, 2.7 mM KCl, 4.3 mM Na_2_HPO_4_, 1.4 mM KH_2_PO_4_, and pH 7.4) solution. The PBMC fraction was obtained by Ficoll-Hypaque centrifugation. The cells were then washed in PBS before culture. The PBMCs were cultured for 24 h at 37°C at a density of 1 × 10^6^ cells/well in Dulbecco's Modified Eagle (DMEM) medium supplemented with 10% (v/v) fetal calf serum (FCS). The viability of PBMCs was measured by trypan blue dye exclusion and was consistently greater than 98%. The cells were then suspended in RPMI-1640 (Invitrogen-Life Technologies).

### 2.3. In Vitro PBMC Stimulation

PBMC were incubated with DMEM containing 10% FCS at 37°C in 5% CO_2_ in 24 well plates (1 × 10^6^ cells/mL). Cells were cultured separately in the presence de 10 *μ*g/mL rMBP::SSP4 protein or medium alone. Cells and culture supernatants were collected at different times (12, 24, 48, 72, and 96 h), cytokine and chemokine concentrations and the expression of genes for cytokines, chemokines, adhesion molecules, and metalloproteinases were determined. All experiments were controlled for stimulation with MBP alone.

### 2.4. Nitric Oxide Measurement

Nitrite accumulation, an indicator of NO synthesis, was measured in the culture medium by Griess reaction [13]. In brief, human PBMC were stimulated with either rMBP::SSP4 (10 *μ*g/mL), MBP (10 *μ*g/mL), LPS from *Escherichia coli* (0.0111:B4, 4 ng/mL) (Sigma Chemical Co), IFN-*γ* (100 U/mL) (Genzyme Diagnostic), or LPS plus IFN-*γ*, respectively. Nonstimulated cells were used as a control. In some cases, N^G^-nitro-_L_-arginine methyl ester (L-NAME; 3 mM) (Sigma Chemical Co) was added separately; similarly 15 *μ*g/mL of Polimyxin B sulphate (PMB) (Sigma Chemical Co.) was added to inhibit the LPS present in the recombinant protein derived from the purification process (data not shown). 100 *μ*L of cell culture medium was mixed with 100 *μ*L of Griess reagent and incubated at room temperature for 15 min. Absorbance at 540 nm was determined, and nitrite concentration was calculated from a sodium nitrite standard curve.

### 2.5. Determination of Cytokine and Chemokine Pattern by ELISA

Interleukin 1-beta (IL-1*β*), IL-6, IL-12, TNF-*α*, IFN-*γ*, and chemokines CCL3, CCL4, CCL5, CXCL10 (IP-10), CXCL8 (IL-8), and CCL11 were quantified by ELISA in culture supernatants of monocyte under different conditions of stimulation, according to the manufacturer's protocol. Briefly, 96-well flat-bottom plates were coated over night with a capture antibody at a final concentration of 2 *μ*g/mL, and then plates were blocked with 10% PBS-FCS, washed three times, and incubated with the cell culture supernatant samples or control antigens overnight at 4°C. After washing, plates were incubated with the respective biotinilated anti-cytokine antibodies (R&D System) at 1 *μ*g/mL for 1 h in the dark. Plates were washed and streptavidin-Alkaline Phosphatase at 1 : 2000 was added for 30 min in the dark then washed, and 100 *μ*L of ABTS (2,2,-azino-bis (3-ethylbenzthiazoline)-6-sulphonic acid) (Zymed) was added as substrate and the reaction was allowed to proceed for 20 min at room temperature (RT); the reaction was stopped with 2% sulphuric acid, and absorbance was read at 415 nm by an ELISA reader (Multiscan MS, Labsystem).

### 2.6. RNA Isolation and RT-PCR

Total RNA from PBMC, cultured in 24-well plates with different treatments for 48 h, was isolated using the TRIzol system (Life Technologies). One microgram of RNA was reverse transcribed to cDNA with an oligonucleotide (poly(dT)16) using the SuperScript II reverse transcriptase (Life Technologies) and the cDNA used as a template for PCR. PCR sequences and PCR conditions used for amplification of GAPDH [17], IL-1*β* [18], IL-6 [18], IL-12p40 [19], IFN-*γ* [19], TNF-*α* [18], CCL3 [19], CCL5 [18], CXCL10 [18], E-selectin [17], ICAM-1 [17], VCAM-1 [17], TNFR-I [20], and TNFR-II [20] were as follows: GAPDH (5′-GGT GAA GGT CGG AGT CAA CGG-3′ and 5′-GGT CAT GAG TCC TTC CAC GAT-3′), IL-1*β* (5′-ATG GCA GAA GTA CCT AAG CTC GC-3′ and 5′-ACA CAA ATT GCA TGG TGA AGT CAG TT-3′), IL-6 (5′-ATG AAC TCC TTC TCC ACA AGC GC-3′ and 5′-GAA GAG CCC TCA GGC TGG ACT G-3′), IL-12p40 (5′-AAC TTG CAG CTG AAG CCA TT-3′ and 5′-TGA TGT ACT TGC AGC CTT GC-3′), IFN-*γ* (5′-GAC CAG AGC ATC CAA AAG A-3′ and 5′-CCT TTT TCG CTT CCC TGT TTT A-3′), TNF-*α* (5′-TTC TGT CTA CTG AAC TTC GGG GT-3′ and 5′-GTA TGA GAT AGC AAA TCG GCT GAC GG-3′), CCL3 (5′-CGC CTG CTG CTT CAG CTA CAC CTC CCG GCA-3′ and 5′-TGG ACC CCT CAG GCA CTC AGC TCC AGG TCG-3′), CCL5 (5′-CGG GAT CCA TGA AGG TCT CCG CGG CA-3′ and 5′-CGG AAT TCC TAG CTC ATC TCC AAA GA-3′), CXCL10 (5′-CCA CGT GTT GAG ATC ATT GCT AC-3′ and 5′-ACA TAG CAC CTC AGT AGA GCT TAC-3′), E-selectin (5′-CTC TGA CAG AAG AAG CCA AG-3′ and 5′-ACT TGA GTC CAC TGA AGC CA-3′), ICAM-1 (5′-TAT GGC AAC GAC TCC TTC T-3′ and 5′-CAT TCA GCG TCA CCT TGG-3′), VCAM-1 (5′-ATG ACA TGC TTG AGC CAG G-3′ and 5′-GTG TCT CCT TCT TTG ACA CT-3′), TNFR-I (5′-TCA GTC CCG TGC CCA GTT CCA CCT T-3′ and 5′-CTG AAG GGG GTT GGG GAT GGG GTC-3′), and TNFR-II (5′- GCT CGC CGG GCC AAT ATG C-3′ and 5′-GGC TTG CAC ACC ACG TCT GA-3′). PCR conditions were as follows: initial DNA denaturation at 94°C for 5 min and 35 rounds of denaturation (95°C for 1 min), annealing (55°C for IL-1*β*, TNF-*α*, IL-6, CCL5, CXCL10, ICAM-1, VCAM-1, and E-selectin, 58°C for TNFR-I, and TNFR-II, 59°C for GAPDH, and 60°C for CCL3, IFN-*γ*, and IL-12p40 for 1 min in each case) and extension (72°C for 1 min). PCR products were electrophoresed on 1.8% agarose gels containing 0.5 *μ*g/mL ethidium bromide and photographed under ultraviolet light. Densitometric analyses were done using the Image J software (Version 1.43 u).

### 2.7. Statistical Analysis

Statistical analysis was performed with GraphPad Prism (Version 5.0). The results are presented as mean ± standard deviation. Analysis of variance (ANOVA) followed by Tukey's post-hoc test was performed to compare the mean values among various groups. A *P* value of <0.05 was considered statistically significant.

## 3. Results

### 3.1. Induction of Nitric Oxide Production by rMBP::SSP4 in PBMCs

**Figure 1:**
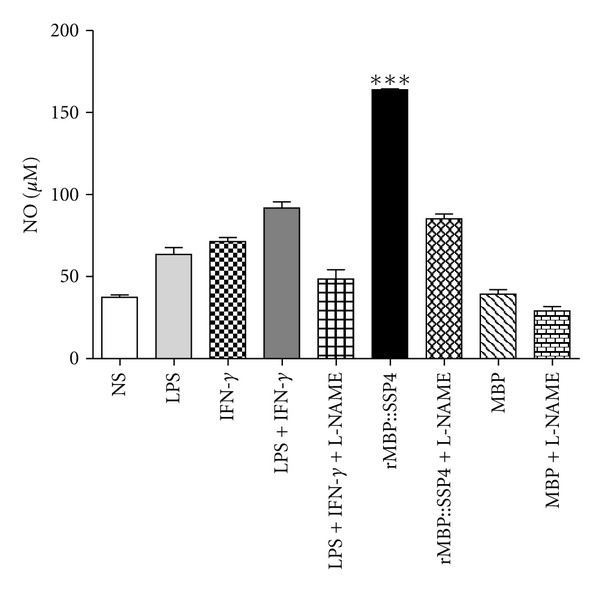
rMBP::SSP4 induces NO synthesis in PBMC. PBMC were stimulated at 48 h with different stimuli. Cells cultured with medium alone were used as controls. Supernatants of cultured cells were harvested, and nitrite concentration was assayed. Data are expressed as means ± SD and are representative of three independent experiments. ***P < 0.0001 compared with non-stimulated cells and controls. NS: non-stimulated.

To first test the ability of the protein rMBP::SSP4 to induce nitric oxide production, PBMCs were stimulated with 10 *μ*g/mL of protein *in vitro*. Result showed that rMBP::SSP4 protein is capable of inducing NO production in PBMC after 48 hours of stimulation (Figure 1), and that production is inhibited by the action of the inhibitor L-NAME. Nitrite values obtained by the stimulation of rMBP::SSP4 protein are statistically significant when compared with the values of nonstimulated cells (*P* < 0.0001) or with the values obtained from cells stimulated with MBP. NO production was increased up to 72 h (data not shown).

### 3.2. rMBP::SSP4 Protein Induces Cytokine and Chemokine Gene Expression

**Figure 2:**
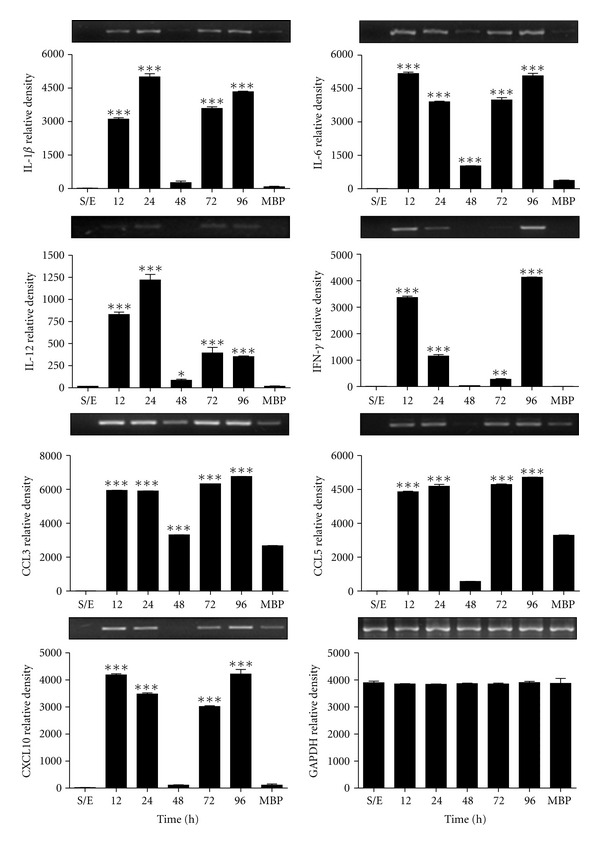
Expression of genes for cytokines and chemokines in PBMC stimulated with rMBP::SSP4. The cells were stimulated with recombinant protein for 12–96 h. The intensities of each band were quantified and plotted from the gels that are on top of each graph corresponding to the expression of genes for cytokines and chemokines. GAPDH was used as control housekeeping gene. ^∗,∗∗,∗∗∗^ *P* < 0.05, 0.001, and 0.0001, respectively, versus unstimulated cells.

Cytokines play a fundamental role during the acute phase of *T. cruzi* infection [21] and affect the function of all cells types involved in an immune response. To investigate whether rMBP::SSP4 protein altered cytokine expression, RT-PCR analysis was performed in PBMC stimulated *in vitro* with rMBP::SSP4 protein (Figure 2). When PBMC were stimulated with rMBP::SSP4 protein, an increase in the expression of genes for IL-1*β*, IL-6, IL-12, IFN-*γ*, CCL3, CCL5, and CXCL10 was observed from 12 to 96 h with low expression at 48 h (Figure 2).

### 3.3. Cytokine and Chemokine Production by PBMC Stimulated with rMBP::SSP4 Protein

**Figure 3:**
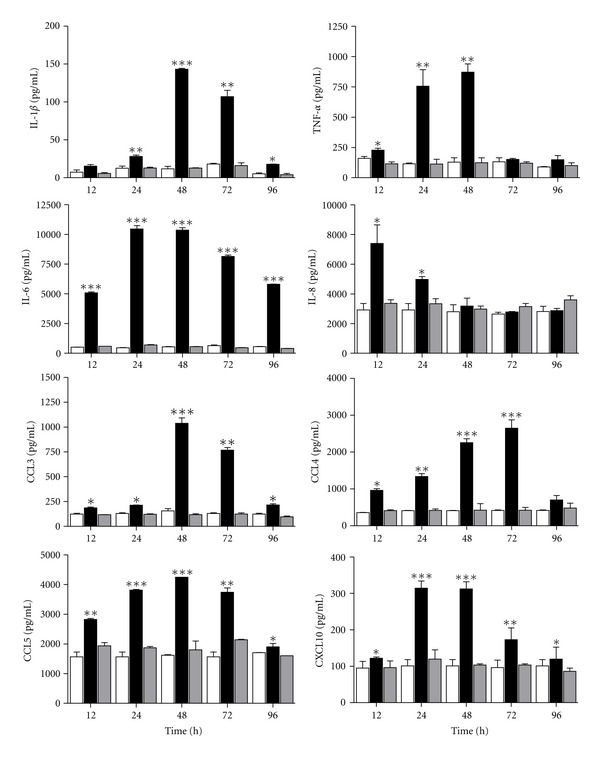
Profile of cytokines and chemokines induced by rMBP::SSP4 in PBMC. PBMC was stimulated with the protein for 12–96 h, and cytokines and chemokines were measured in cells' culture supernatants by ELISA. Unstimulated cells (open bars), cells stimulated with rMBP::SSP4 (full bars), and cells stimulated with MBP (gray bars). Histograms show values in pg/mL (means ± SD) of three experiments run in duplicate. ^∗, ∗∗, ∗∗∗^ *P* < 0.05, 0.001, and 0.0001, respectively, versus unstimulated cells.

When PBMCs were stimulated with rMBP::SSP4 protein, the production of IL-1*β*, TNF-*α*, and IL-6 significantly increased (Figure 3). For IL-1*β*, the increase was observed at 24–72 h, while for TNF-*α*, the increase was from 12–48 h and a sustained production of IL-6 from 12–96 h of interaction, with a maximum production at 24–48 h. Likewise, we observed an increase in the production of chemokines, such as IL-8, CCL3, CCL4, CCL5, and CXCL10 in PBMC stimulated with rMBP::SSP4 protein. Production of IL-8 was observed only from 12 to 24 h of interaction, and an increase in the production of CCL3, CCL5, and CXCL10 with a maximum production at 48 h and decreased at 96 h of interaction. CCL4 production was also observed with a peak of synthesis at 72 h (Figure 3).

### 3.4. rMBP::SSP4 Protein Induces Genes of Adhesion Molecules and TNF-Receptors

**Figure 4:**
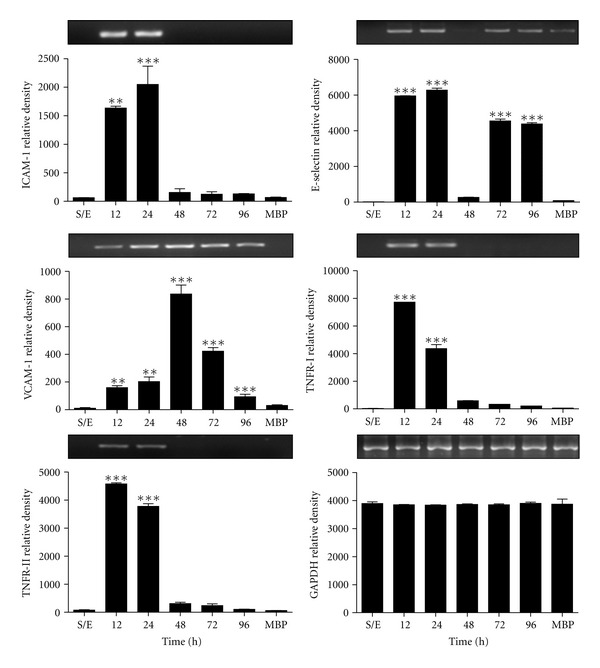
Effect of rMBP::SSP4 on gene expression of CAMs and TNFRs in PBMCs. RT-PCR analysis of CAMs and TNFRs mRNAs in PBMCs was performed as described (see Section 2). PBMCs were stimulated with the protein for 12–96 h, S/E (nonstimulated). The intensities of each band were quantified and plotted from the gels that are on top of each graph corresponding to the expression of genes. GAPDH was used as a control housekeeping gene. ^∗∗, ∗∗∗^ *P* < 0.001 and 0.0001, respectively, versus unstimulated cells.

To investigate whether rMBP::SSP4 protein was able to induce gene expression of CAMs and TNF receptors, PBMCs were stimulated with recombinant protein. We observed in PBMC an increased expression of gene for ICAM-1 (12–24 h) and an increase in the expression of genes for E-selectin and VCAM-1, with a maximum expression at 96 and 48 h, respectively. Likewise, we observed an increase in the expression of genes for TNFR-I and TNFR-II of the 12 to 24 h (Figure 4).

## 4. Discussion

Parasitic infections are prevalent in both tropical and subtropical areas. Most of the affected and/or exposed populations are living in developing countries where control measures are lacking or inadequately applied. Although significant progress has been made in our understanding of the immune response to parasites, no definitive step has yet been successfully done in terms of operational vaccines against parasitic diseases [22]. Pathophysiology of Chagas' disease is not completely defined, although innate and adaptive immune responses are crucial. In acute infection, some parasitic antigens can activate macrophages, and this may result in proinflammatory cytokine production, nitric oxide synthesis, and consequent control of parasitemia and mortality [23]. During the acute phase of infection, *T. cruzi *replicates extensively and releases immunomodulatory molecules that delay parasite-specific responses mediated by effector T cells. This mechanism of evasion allows the parasite to spread in the host [10]. The disturbed cytokine-chemokine network could play an important role in the onset of diseases with inflammatory processes [24]. We investigated whether rMBP::SSP4 protein induced NO production and cytokine gene expression in PBMC. The results showed that rMBP::SSP4 protein induced NO production in PBMC from 24 to 48 h (Figure 1). We have previously shown that rMBP::SSP4 protein was able to induce nitric oxide (NO) production by spleen and peritoneal macrophages (pM*ϕ*s) and macrophages from immunized mice [13]. Inhibition of NO production by L-NAME in murine M*ϕ*s, results in a down-regulation of *i*NOS expression [25]. Our results showed that NO production was affected when stimulated PBMCs were incubated in the presence of L-NAME, thus indicating that the enzyme *i*NOS was participating in NO synthesis, it is also known that TNF-*α* regulates NOS expression and/or activity, which exerts direct effects on NO production [26]. According to these observations, and the fact that rMBP::SSP4 protein induces NO production by PBMC, the participation of NO in the suppression of T cell activation has been reported in a number of biological systems. In the case of *T. cruzi*, previous studies have shown that IFN-*γ* and nonoxidative molecules (TNF-*α* and NO) could play a role in the control of *T. cruzi* infection in mice [27, 28]. Furthermore, a series of experiments supports the notion that IFN-*γ* and TNF-*α* mediated activation of macrophages, which leads to increased production of NO, and in turn suppresses T cell activation. Therefore, it is likely that NO production during the initial phase of acute infections might participate in the clearance of parasites by macrophages, whereas its overproduction during the late phase of acute infection would account for the immunosuppression observed [21].

We investigated the cytokine and chemokine gene expression pattern in these cells as well as the production of these molecules in the culture supernatant. Results showed that this antigen induces the secretion of several chemokines (IL-8, CCL3, CCL4, CCL5, and CXCL10) and cytokines, such as IL-1*β*, IL-6, IFN-*γ*, and TNF-*α* in considerable amounts, whereas IL-12 was produced at a very low level suggesting that SSP4 is an immunomodulatory molecule of *T. cruzi.* Furthermore, IFN-*γ* is an important Th1 proinflammatory cytokine that could participate in the generation of Tregs cells [29] during acute phase, thus as has been observed in the mouse model using rMBP::SSP4 [10]. In addition, IL-12 has been described to have stimulatory effects on hematopoietic precursor cells and on B lymphocytes. The IL-12 produced during this inflammatory phase, both by direct action and, indirectly, by determining the composition of the cytokine milieu at the site of the immune response, induces differentiation of T helper type 1 (Th1) cells while inhibiting the generation of Th2 cells. Thus, because of its double function of a proinflammatory cytokine and an immunoregulatory factor, IL-12 plays a key role in the resistance to infections, particularly those mediated by bacteria or intracellular parasites, against which phagocytic cell activation and Th1-mediated responses are particularly effective. However, because of the same activities, IL-12 also plays a role in pathological situations, such as septic shock, tissue damage during inflammation, and organ-specific autoimmune diseases [30]. Accordingly, there are reports in animal models showing that inflammatory cytokines play a central role in acute *T. cruzi* infection; upon activation, such cells secrete proinflammatory cytokines and chemokines are promptly released and further activate other inflammatory cells [31]. This pattern of expression has been observed in the inflammatory responses in cardiomyocytes during *T. cruzi* infection. It was shown that heart tissues collected from *T. cruzi*-infected rats expressed IL-6, IL-1*β*, TNF-*α*, and *i*NOS, moreover, hearts of mice infected and cardiomyocytes express the same pattern of cytokines and chemokines [4, 32–34].

SSP4 superficial protein is expressed shortly after trypomastigotes begin to transform into amastigotes, in a phase which is released the amastigote-specific SSP4 protein [12], this protein can interact with and activate PBMC, secreting cytokines, chemokines, NO, and other molecules which might attract leukocytes to the inflammatory site after interaction with specific molecules of the parasite, and that rMBP::SSP4 can significantly increase this effect. Since it has been observed that PBMC recruitment is a rapid and remarkable phenomenon during acute infection with the intracellular protozoan parasite *T. cruzi*, the causative agent of Chagas' disease, the functional capabilities of these cells during the infection, however, are poorly understood [35]. The ability of monocyte-derived macrophages to process and present antigens, produce cytokines, and provide costimulatory signals demonstrate their pivotal role in initiating immune responses [36]. Activated macrophages exert critical activities in immunity to parasites, playing a key role in the mechanism for halting the acute *T. cruzi* infection. Activation of macrophages by parasite antigens results in proinflammatory cytokine production and consequent control of parasitemia and mortality [24]. On the other hand, it has been observed that this protein induces high production of IL-6 [13, 15], according to our results and because of the pleiotropic character of IL-6 has made it difficult to obtain a clear answer for the role of this cytokine in this model; however, the production of IL-6 observed in PBMCs could possibly modulate the differentiation of T cells infiltrated through the process of chemotaxis toward a Th2 pattern [37] and may later be involved in the maturation process of B cell [13], during polyclonal activation observed in the acute phase of infection [38]. This inflammatory T cell and antibody response leads to control—but not complete elimination—of tissue and blood parasitism.

We showed that the expression of adhesion molecules and TNF receptors are upregulated in PBMC by the stimulation *in vitro* with rMBP::SSP4 protein (Figure 4). Accumulation of leukocytes at the site of local injury or infection of endothelial cells is dependent on the interaction of circulating leukocytes with vascular adhesion molecules, such as E-selectin, VCAM-1, and ICAM-1 [39]. Likewise, it is known that TNF receptors play a role in inflammatory process; TNFR-I may have anti-inflammatory and inflammatory effects, depending on the signaling pathway that is activated. TNFR-II is associated mainly with inflammatory and antiapoptotic processes [40]. Expression of TNFR-II observed in PBMC suggested in the context of infection that parasite probably ensures the survival of the cell to perpetuate the process of infection and their tissue retention, possibly promoted by the action of IL-8 [41].

The soluble parasite factors can elicit a complex series of cellular interactions leading to an immunosuppression state, in addition, may have additional roles in driving early immunological events toward Th2-type or anti-inflammatory responses fully polarized. These raise the distinct possibility that the production of parasite factors that interact with cell surface receptors may be one mechanism, whereby the parasite is able to interfere with the regulation of the induction/initiation phase of the host immune response that may protect the host from excessive inflammation and may potentiate the parasite's own survival [8].

Finally, inflammatory response that follows the infection with *T. cruzi* is essential for host resistance to infection but is also responsible for the diverse pathology observed in Chagas disease [4]. Parasite persistence depends on a combination of factors, including release of molecules that interfere with the immune response. Therefore, suppression induced by parasite molecules is more relevant at the acute phase, when the concentration of such molecules can be fairly high. Although the amastigote stage is considered essentially as the stage of intracellular replication, this form of the parasite is present in the circulation during the acute phase of infection in mice and can enter and develop in both mammalian phagocytic cells (*in vitro*) and nonmammalian phagocytic cells [11].

In conclusion, all these results suggest that the amastigote SSP4 molecule could play a key role in the inflammatory process, modulating the expression and production of inflammatory molecules, which may represent a mechanism participating in the immunoregulatory and/or immunosuppressive processes carried out by *T. cruzi* during the development of the acute phase of Chagas' disease.
